# Supplementary material for: Enhanced feedback performance in off-resonance AFM modes through pulse train sampling
Source: Beilstein J Nanotechnol. 2024 Feb 1;15:134–43. doi: 10.3762/bjnano.15.13 (PMC10840551; doi:10.3762/bjnano.15.13)
Supplement: File 1 — Simulations on unity feedback closed-loop ORT controller and closed-loop bandwidth measurements of both methods. [file Beilstein_J_Nanotechnol-15-134-s001.pdf]

BEILSTEIN JOURNAL OF NANOTECHNOLOGY

Supporting Information

for

Enhanced feedback performance in off-resonance AFM modes through pulse train sampling

Mustafa Kangül, Navid Asmari, Santiago H. Andany, Marcos Penedo and Georg E. Fantner

Beilstein J. Nanotechnol. **2024,** *15,* 134–143. doi:10.3762/bjnano.15.13

Simulations on unity feedback closed-loop ORT controller and closed-loop bandwidth measurements of both methods

License and Terms: This is a supporting information file under the terms of the Creative Commons Attribution License (https://creativecommons.org/licenses/by/4.0). Please note that the reuse, redistribution and reproduction in particular requires that the author(s) and source are credited and that individual graphics may be subject to special legal provisions.
The license is subject to the *Beilstein Journal of Nanotechnology* terms and conditions: (https://www.beilstein-journals.org/bjnano/terms)

1. Simulations on unity feedback closed-loop ORT controller

Figure S1A illustrates the controller in a unity gain closed-loop feedback. The integral gain ensures zero steady-state error and, together with the delay element, forms a second-order transfer function which we provide in-detail analysis of in this manuscript. For simplicity, our theoretical calculations and experimental verification include only the integral gain but not the proportional gain, even though propotional gain would be helpful for better tracking. We have performed theoretical simulations on MATLAB based on Equation 2 which is derived by the following set of equations.

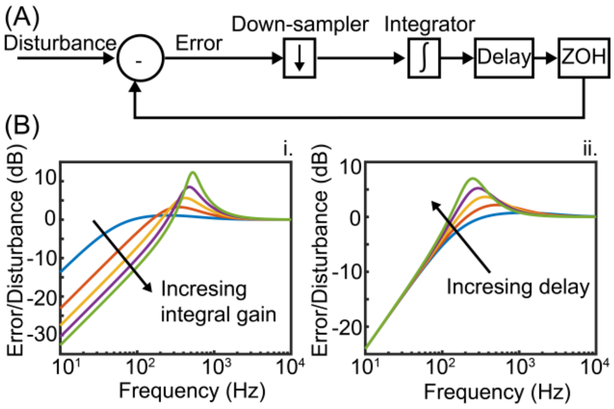

Figure S1: The effect of I gain and delay on a simplified ORT controller (A). Error rejection is plotted for 1 kHz ORT rate with constant delay – increasing gain (B) i. and constant gain – increasing delay (B) ii.

discrete domain integrator (accumulation):

$$\frac{k_i}{1 - z^{-1}} \quad \text{(s1)}$$

discrete time operator:

$$z = e^{sT_{ort}} \quad \text{(s2)}$$

delay element:

$$e^{-sT_{delay}} \tag{s3}$$

zero order hold:

$$\frac{1-e^{-sT_{ort}}}{sT_{ort}} \tag{s4}$$

open loop transfer function:

$$G(s) = \frac{k_i}{1-e^{-sT_{ort}}} \times e^{-sT_{delay}} \times \frac{1-e^{-sT_{ort}}}{sT_{ort}} = \frac{k_i}{sT_{ort}} e^{-sT_{delay}} \tag{s5}$$

first order Padé approximation:

$$e^{-sx} = \frac{1-\frac{sx}{2}}{1+\frac{sx}{2}} \tag{s6}$$

Simplification of the open-loop transfer function:

$$G(s) = \frac{k_i}{sT_{ort}} \times \frac{1-\frac{sT_{delay}}{2}}{1+\frac{sT_{delay}}{2}} \tag{s7}$$

closed-loop transfer function:

$$H(s) = \frac{1}{1+G(s)} = \frac{1}{1+\frac{k_i}{sT_{ort}} \times \frac{1-\frac{sT_{delay}}{2}}{1+\frac{sT_{delay}}{2}}} = \frac{sT_{ort} + \frac{s^2}{2} T_{ort} T_{delay}}{k_i + s\left(T_{ort} - \frac{k_i T_{ort}}{2}\right) + s^2\left(\frac{T_{ort} T_{delay}}{2}\right)} \tag{s8}$$

The frequency response of error versus the external disturbance plot displayed in Figure S1 (B) shows the effect of the delay and the integral gain on the resulted error signal. In

the simulations depicted on the left panel, we increase the integral gain keeping the ORT period and delay constant at 1 ms and 0.5 ms, respectively. Increasing the integral gain decreases the error for a given frequency. However, it also increases the magnitude and the quality factor of the resonance peak due to the delay. Especially, in slow ORT applications, the zero-order hold delay of half an ORT period is the dominating element in the overall delay of the closed-loop system. On the second simulation set, right panel in Figure S1 (B), we increase the delay by keeping the integral gain constant. Increasing the delay does not significantly change the disturbance rejection at low frequencies, but it creates a resonance peak with a higher amplitude, as seen in Figure S1 (B)ii. To prevent triggering closed-loop oscillations due to a resonance peak, we have to decrease the integral gain and hence degrade disturbance rejection. Simulations based on Equation 1 show that the ORT period and the delay of zero order hold impose a limit for the I gain, which reduces tracking quality and forces the users to image slower.

2. Closed-loop bandwidth measurements of both methods

The functionality of the proposed method is assessed in a test framework, where we have conducted disturbance rejection measurements using a lock-in amplifier (Anfatec eLockIn 205), as illustrated in Figure S2. The reference output of the lock-in amplifier is added to the Z output of the controller as the disturbance and fed to the controller as the deflection input. This is equivalent to replacing the physical AFM with a unity gain component, where the lock-in amplifier reference output represents the topography of the sample. The ORT rate is set at 1 kHz and the interaction window is selected as 12.8% of the period. Integral gains are set at the highest possible value such that the system does not experience an oscillatory behavior. The error that is recorded during the interaction window is sent to the

lock-in amplifier as the input signal. The frequency response of the system is generated by sweeping the reference frequency of the lock-in amplifier and recording the corresponding error, as plotted in Figure 2 (D). For the impulse sampling mode, the remaining error peaks around 500 Hz, which is the Nyquist frequency due to the 1 kHz ORT rate. Using a higher I gain causes an increase in the amplitude of the peak which triggers an oscillation in the closed-loop system. However, for the pulse sampling mode, the effective integral gain can be set higher due to the significantly lower delay time in the closed-loop, reducing around 40 dB the error for the same disturbance. This experiment demonstrates that employing multiple sampling points per ORT curve and reducing the delay leads to improved tracking performance.

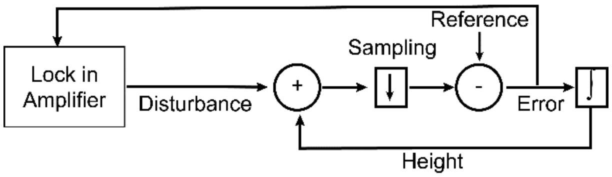

Figure S2: Tracking quality of the closed-loop controller is measured by the ratio of recorded error and introduced disturbance with the illustrated setup. Reference output of the lock in amplifier is added to the height signal generated by the controller as the disturbance. The calculated error in the closed is sent to the lock in amplifier as the input.
